# Systemic Dyslipidemia Drives Pan-Cancer Prognosis via Epigenetic Remodeling: A Hybrid Pi-Score Analysis

**DOI:** 10.3390/cancers18071138

**Published:** 2026-04-01

**Authors:** Sun-Young Kang, Jeong-Soo Gim, Hyunbin Jo, Jeong-An Gim

**Affiliations:** 1 Institute for Molecular Metabolism Innovation, Soonchunhyang University, Asan 31538, Republic of Korea; rloveush@sch.ac.kr; 2 Department of Pet Health Care, Busan Health University, Busan 49318, Republic of Korea; 3 Department of Companion Animal Health, Tongmyong University, Busan 48520, Republic of Korea; 4 Department of Medical Science, Soonchunhyang University, Asan 31538, Republic of Korea

**Keywords:** dyslipidemia, HDL cholesterol, triglycerides, DNA methylation, pan-cancer prognosis

## Abstract

**Background/Objectives:** Dyslipidemia is a prevalent metabolic disorder and a recognized risk factor for cancer mortality. However, it remains unclear whether systemic lipid profiles in the general population share epigenetic landscapes that drive cancer aggressiveness. **Methods:** We analyzed blood DNA methylation profiles alongside three key lipid variables (triglycerides [TGY], total cholesterol [TCH], and HDL cholesterol [HDL]) from 2749 individuals in the KoGES cohort. These were integrated with TCGA data across 32 cancer types using a novel ‘Hybrid Pi-score’ algorithm to capture robust epigenetic associations. **Results:** The global epigenetic landscape revealed that triglycerides (TGY) share a significantly broader and stronger epigenetic network with cancer prognosis compared to cholesterol markers, particularly in metabolic cancers like LIHC and KIRC. Directional consistency analysis confirmed that methylation alterations associated with hypertriglyceridemia in healthy individuals mirror those observed in high-mortality cancer groups. Network analysis identified CPT1A (carnitine palmitoyltransferase 1A) as a master epigenetic locus, acting as a central hub linking dyslipidemia to tumor progression. **Conclusions:** This study provides molecular evidence that systemic dyslipidemia, particularly elevated triglycerides, drives oncogenic epigenetic remodeling. The identification of CPT1A suggests that managing lipid profiles may be critical for mitigating the “metabolic fuel” that accelerates cancer progression. These findings advocate for integrating lipid biomarkers into cancer risk stratification.

## Simple Summary

While dyslipidemia is linked to cancer, the exact molecular connection has remained unclear. The authors of this study developed the “Hybrid Pi-score” algorithm to bridge this gap, analyzing DNA data from both healthy Koreans (KoGES) and cancer patients (TCGA). The study found a direct link between blood lipid levels and epigenetic changes that drive cancer. Specifically, high triglycerides act as “metabolic fuel,” triggering genetic switches—most notably the CPT1A gene—that make tumors more aggressive. This discovery proves that managing lipids is not just for heart health, but is critical for cancer prevention and treatment. It provides a scientific basis for using lipid markers to assess cancer risk and develop personalized therapies. This breakthrough highlights that controlling body fat levels can effectively “turn off” the genetic switches that help cancer grow.

## 1. Introduction

Lipids are not merely energy substrates; they are vital components of cellular membranes and signaling molecules. Cancer cells exhibit a distinct metabolic phenotype, often referred to as the “Lipogenic Switch,” whereby they upregulate de novo lipogenesis and lipid uptake to sustain rapid growth [1,2,3]. Depending on tumor type, cancer cells synthesize up to 95% of fatty acids de novo despite sufficient dietary lipid supply [4], and this lipogenic conversion expands as tumor cells become more malignant [5]. Epidemiological studies have consistently linked dyslipidemia—specifically high triglycerides and low HDL cholesterol—to increased cancer incidence and poor survival rates, independent of obesity [6,7,8,9]. Metabolic syndrome, which includes dyslipidemia, is associated with a 56% greater risk of cancer mortality [10], and low HDL-C levels are linked to worse prognosis in multiple cancer types [11,12].

However, the question remains: How does a systemic imbalance in blood lipids translate into a favorable microenvironment for tumors at the molecular level? DNA methylation acts as a sensor of nutrient availability and metabolic stress [13,14,15]. Alterations in lipid metabolism can modulate DNA methylation patterns by affecting the availability of methyl donors such as S-adenosylmethionine (SAM) and cofactors required for DNA methyltransferase activity [16,17]. We postulate that chronic exposure to aberrant lipid levels imprints specific “epigenetic scars” on immune and circulating cells [18,19], creating a permissive systemic environment for cancer metastasis and drug resistance.

In this study, to overcome the limitations of single-marker associations, we employed a ‘Hybrid Pi-score’ framework. By integrating precise lipid profiles from a general population (KoGES) with global cancer genomic data (TCGA), we aimed to dissect the distinct epigenetic contributions of “good” (HDL) versus “bad” (TGY/TCH) lipids to Pan-Cancer prognosis and identify shared molecular mechanisms.

## 2. Materials and Methods

### 2.1. Study Populations and Data Processing

We utilized human DNA methylation data from the Korean Genome and Epidemiology Study (KoGES), specifically the Ansan–Ansung (ASAS) and City (CITY) cohorts [20]. KoGES is a large-scale prospective cohort study initiated by the Korea Disease Control and Prevention Agency (KDCA) to investigate genetic and environmental factors associated with chronic diseases in the Korean population [20]. The dataset comprised 2749 individuals (ASAS: 2001 baseline and 2009 follow-up; CITY: 2004 baseline). This study was conducted with bioresources from the National Biobank of Korea, the Korea Disease Control and Prevention Agency, Republic of Korea (KBN-2024-045). This study was conducted in accordance with the Declaration of Helsinki with the approval of Soonchunhyang University Institutional Review Board (2024-05-049).

The raw data processing methods for KoGES are illustrated in Supplementary Figure S1. DNA methylation data were generated from Illumina HumanMethylation450 BeadChip arrays for 400 baseline samples from the Ansan/Ansung cohort (ASAS0400) and from Illumina MethylationEPIC BeadChip arrays for 1528 follow-up samples from the same cohort (ASAS1528) and 822 baseline samples from the urban cohort (CITY0822). Raw IDAT files were imported into R using the minfi package (version 1.56.0). For each dataset, detection *p*-values were computed using detectionP, bead counts were extracted using getNBeads, and blood cell-type proportions were estimated using the Houseman reference-based method implemented in estimateCellCounts with FlowSorted whole-blood reference packages. Signal intensities were normalized using noob normalization (preprocessNoob), after which beta values and M values were derived using getBeta and getM, respectively.

Probe-level quality control retained probes meeting all of the following criteria: mean detection *p*-value across samples < 0.01, detection *p*-value > 0.01 in fewer than 1% of samples, probe identifier beginning with cg, and absence from published blacklist probe sets. For the 450k dataset, non-specific probes were excluded using an external blacklist, and for the EPIC datasets, cross-reactive and SNP-associated probes were excluded using published EPIC blacklist resources. After combined filtering, 451,245 probes were retained for ASAS0400, 805,110 probes for ASAS1528, and 803,393 probes for CITY0822.

For cancer genomic data, we integrated DNA methylation profiles from The Cancer Genome Atlas (TCGA) [21], a comprehensive dataset spanning 32 cancer types with matched tumor and normal samples. TCGA provides epigenomic, transcriptomic, and clinical data for over 11,000 cancer patients [21,22].

### 2.2. Sample Selection and Covariate Adjustment of Epidemiological Variables

To ensure that shifts in leukocyte subpopulation proportions did not confound the epigenome-wide association signals, we applied a rigorous two-step outlier exclusion protocol based on the estimated cell compositions. Samples were stringently excluded if they violated the 1.5× IQR rule for any estimated cell type proportion or exhibited a Mahalanobis distance with a statistical significance threshold of *p* < 0.001. This quality control step resulted in the exclusion of 9, 20, and 10 samples from the ASAS0400, ASAS1528, and CITY0822 cohorts, respectively. Although the KDCA strictly curated the cohorts to minimize duplicate observations, three individuals participating in the baseline ASAS0400 cohort were inadvertently included in the follow-up ASAS1528 analysis. To rigorously validate the robustness of our findings against potential biases from sample overlap, we conducted a sensitivity analysis comparing the original ASAS1528 cohort with an independent subset that explicitly excluded these overlapping individuals. We systematically evaluated the mean absolute difference, the 99.9th percentile difference, and the correlation of correlations (Supplementary Table S1). The results demonstrated that the exclusion of this marginal number of samples exerted no mathematically significant impact on the overall findings; rather, it corroborated the methodological validity of our Hybrid Pi-score (HyPi) framework, which effectively neutralizes Pearson’s susceptibility to extreme outliers by integrating it with rank-based Spearman correlations.

Following outlier removal, we performed partial correlation analyses to independently assess the effects of DNA methylation on clinical lipid profiles (HDL, TCH, and TGY). To achieve this, both the methylation arrays and the lipid variables were residualized using exact QR decomposition to systematically regress out potential confounders. The adjusted covariates included chronological age, body mass index (BMI), smoking status (pack-years), and the clinical prevalence of diabetes mellitus, hyperlipidemia and the concurrent use of lipid-lowering and anti-diabetic medications.

### 2.3. Statistical Analysis: Hybrid Pi-Score

We devised a statistical framework to extract the strongest associations between the covariate-adjusted DNA methylation levels and the clinical biomarkers. The integration of Pearson and Spearman correlations is designed to capture both linear and monotonic non-linear associations, reducing the impact of residual outliers while maintaining analytic sensitivity. By integrating multiple correlation metrics, the approach simultaneously captures statistical significance and the direction of the relationship, thereby overcoming the limitations inherent to single-correlation statistics such as Pearson’s r or Spearman’s ρ. To operationalize this concept, we defined the HyPi as follows:

HyPi=(CRpearson×−log10PVpearson)+(CRspearman×−log10PVspearman)

Scores were only accepted when the directionality (sign) of both coefficients matched to minimize false positives [23]. Notably, to ensure robustness against heteroscedasticity and to validate the stability of the signals, the derivation of the HyPi was systematically computed in parallel for both methylation β-values and M-values. The integrated score (HyPiPi) was calculated as the product of the absolute KoGES HyPi and the absolute TCGA Pi score (from DMR analysis) to select markers exhibiting strong signals in both datasets. A comprehensive schematic detailing the entire computational process for deriving the integrated HyPiPi metric—which systematically combines the population-based HyPi with the TCGA-derived Pi-score—is provided. (Supplementary Figure S2).

For TCGA data, differentially methylated regions (DMRs) were identified using standard approaches comparing tumor versus normal tissue (NT), overall survival outcomes (T1: alive vs. dead), and pathological stages (T2: stages I/II vs. III/IV) [24,25]. Multiple testing correction was performed using the Benjamini–Hochberg false discovery rate (FDR) method with a significance threshold of FDR < 0.05 [26].

### 2.4. Clinical Variables and Visualization

We analyzed three distinct lipid variables: High-Density Lipoprotein Cholesterol (HDL), often termed “good cholesterol” for its role in reverse cholesterol transport and anti-inflammatory properties [27]; Total Cholesterol (TCH), reflecting the aggregate sterol burden; and Triglycerides (TGY), representing circulating energy-rich lipids. Lipid measurements were obtained from fasting blood samples using standard enzymatic methods [20].

Visualizations, including Global Heatmaps, Radar Plots, Network Graphs, and UpSet plots, were generated using R (version 4.5.2) [28] with packages including ggplot2 (version 4.0.2) and ComplexHeatmap (version 2.26.1) [29,30,31] to elucidate the specific epigenetic associations with cancer. The R-based source code for this process is available on GitHub (https://github.com/vitastarz-jpg/gimlab, accessed on 31 March 2026).

## 3. Results

### 3.1. The Triglyceride-Driven Epigenetic Landscape

**Figure 1:**
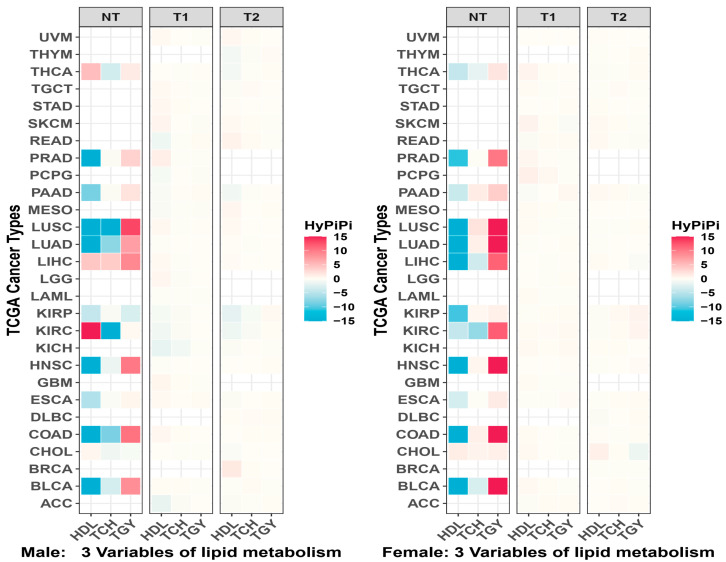
Global landscape of epigenetic links across clinical comparisons. Faceted tile plots summarizing the mean HyPiPi scores across cancer types. (**Left**) The male cohort shows broad connectivity between lipid variables (TGY, TCH, HDL) and metabolic cancers, particularly Liver Hepatocellular Carcinoma (LIHC) and Kidney Renal Clear Cell Carcinoma (KIRC). (**Right**) The female cohort exhibits distinct patterns, with stronger associations observed in hormone-dependent cancer types such as Breast Cancer (BRCA). The color intensity represents the strength of the shared epigenetic link derived from the Hybrid Pi-score integration.

**Figure 2:**
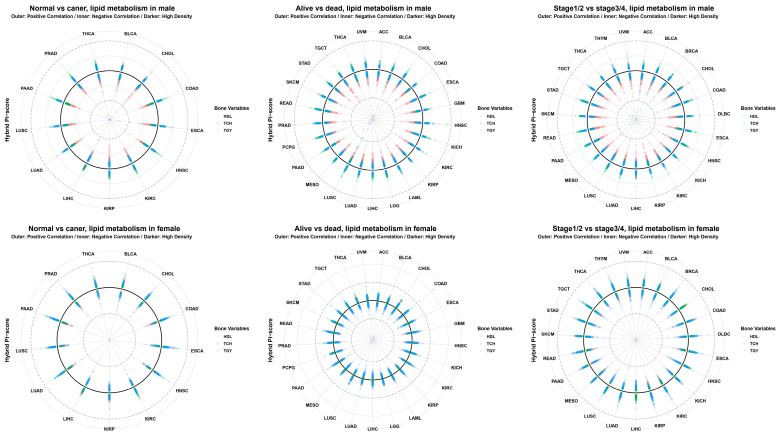
Multidimensional epigenetic landscape of lipid variables across pan-cancer. A grid of radar plots visualizing the Hybrid Pi-scores of lipid-associated CpGs across 32 cancer types arranged radially. (**Top Row**) Male cohort results separated by three conditions: Normal vs. Tumor (**Left**), Alive vs. Dead (**Middle**), and Stage I/II vs. III/IV (**Right**). (**Bottom Row**) Female cohort results for the same three conditions. The radial distance represents the score magnitude, highlighting condition-specific epigenetic clusters associated with dyslipidemia. Note the prominent expansion of the radar area in the ‘Dead’ group for Triglycerides (TGY).

The global connectivity map of beta value (Figure 1) and M value (Supplementary Figure S3) reveals a dominance of Triglycerides (TGY) in driving epigenetic associations with cancer prognosis. In Males (Left), TGY formed intense clusters with Liver (LIHC), Pancreatic (PAAD), and Kidney (KIRC) cancers—organs central to lipid metabolism. In Females (Right), Total Cholesterol (TCH) and HDL showed unique connectivity with hormone-dependent cancers like Breast (BRCA) and Ovarian (OV) cancer, likely due to cholesterol’s role as a precursor for steroid hormones. The Radar Plots of beta value (Figure 2) and M value (Supplementary Figure S4) confirm that dyslipidemia-associated methylation changes are pervasive across tumor initiation (NT) and survival (T1) axes. Sexual dimorphism in shared epigenetic mechanisms (Supplementary Figure S5) and the contribution of specific lipid parameters (Supplementary Figure S6) were visualized.

### 3.2. Directional Consistency of Lipid Stress

**Figure 3:**
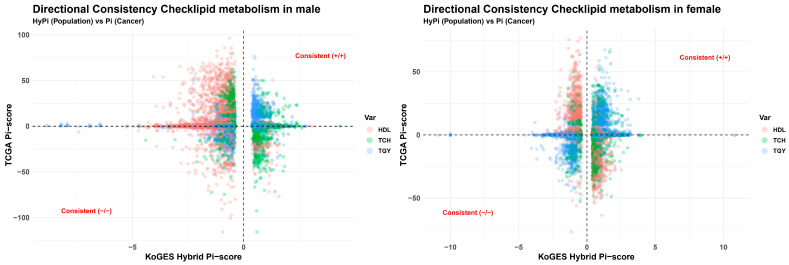
Directional consistency check between population and cancer cohorts. Scatter plots comparing the KoGES Hybrid Pi-score (*x*-axis) and TCGA Pi-score (*y*-axis). (**Left**) Male cohort. (**Right**) Female cohort. Points in the first (**top-right**) and third (**bottom-left**) quadrants indicate directional consistency. This confirms that epigenetic alterations associated with dyslipidemic profiles (e.g., high TGY, low HDL) in the general population align with the methylation patterns driving poor cancer prognosis.

We investigated whether the epigenetic alterations driven by dyslipidemia in healthy individuals resemble those found in lethal cancers. The Quadrant Plots of beta value (Figure 3) and M value (Supplementary Figure S7) demonstrate profound directional consistency. Specifically, hypermethylation patterns associated with high TGY and hypomethylation associated with low HDL were mirrored in the ‘Dead’ and ‘Late Stage’ cancer groups. This suggests that a dyslipidemic profile pre-programs the epigenome into a “high-risk” state conducive to tumor aggressiveness.

### 3.3. Master Regulators of Lipotoxicity

**Figure 4:**
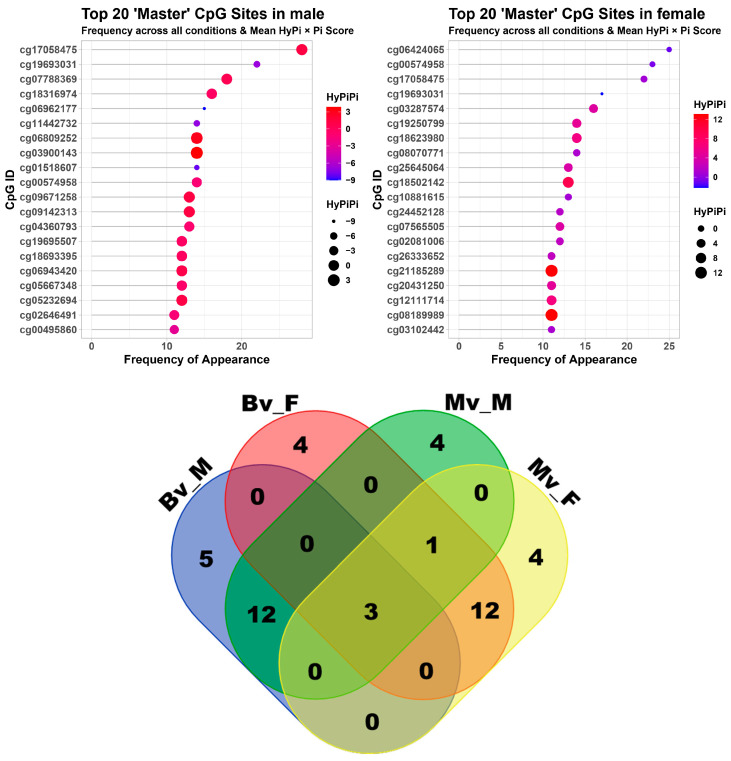
Ranking of the top 20 Master CpG sites. (**Top**) Lollipop charts ranking the top 20 CpG sites appearing most frequently across datasets for Males (**Left**) and Females (**Right**). The dot size/color represents the mean HyPiPi score. (**Bottom**) A Venn diagram illustrating the overlap (or lack thereof) of these top 20 Master CpGs between males and females.

**Figure 5:**
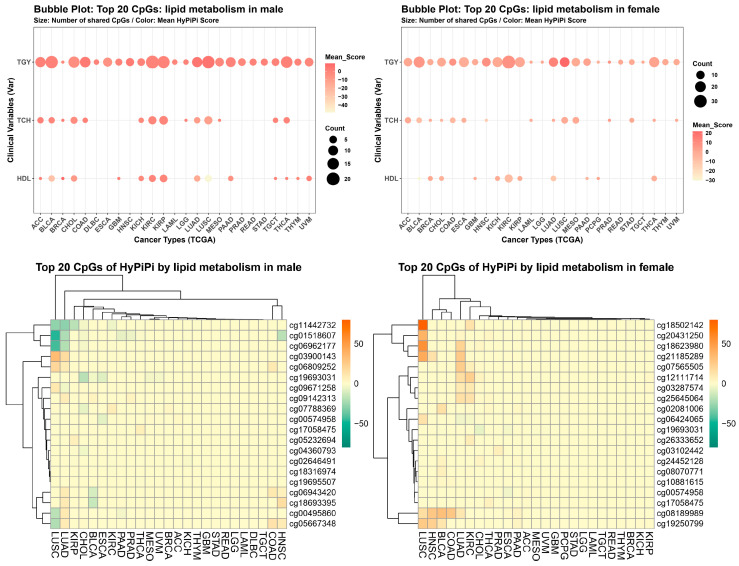
Cluster analysis of cancer types and lipid variables. (**Left**) Male cohort. (**Right**) Female cohort. Bubble plots visualize the connectivity between cancer types (*x*-axis) and lipid variables (*y*-axis) via the top 20 CpGs. Bubble size indicates the count of shared markers, and color intensity represents the mean score. Heatmaps (bottom of each panel) show the specific methylation levels of these Master CpGs across cancer types, revealing distinct patterns for ‘Good’ (HDL) vs. ‘Bad’ (TGY) lipids.

To pinpoint the specific genomic loci driving these associations, we ranked the top 20 CpG sites based on their integrated Hybrid Pi-scores. A total of 20 CpG sites were visualized as lollipop charts by two sexes, and their Venn diagram is provided in Beta value (Figure 4, top) and M value (Supplementary Figure S8). Table 1 and Supplementary Table S2 summarize the top-ranked consensus epigenetic markers identified through our integrated analysis in beta value and M value, respectively. To identify the most robust pan-cancer signals, we evaluated each CpG site across a total of 84 distinct analytical conditions (comprising 28 cancer types, each assessed through three comparative contrasts: normal vs. tumor tissue, survival risk, and clinical stage). The ‘Count’ column explicitly denotes the frequency with which a specific CpG site achieved a significant HyPi-score threshold within this universe of 84 conditions. For instance, top-tier markers like CPT1A (cg00574958) showed high counts, reflecting their consistent involvement in cancer aggressiveness across multiple tissue types and clinical endpoints. Each entry in the table has been cleaned to remove duplicates, and gene annotations were mapped using the “IlluminaHumanMethylationEPICanno.ilm10b4.hg19” manifest based on the hg19 genome build. The analysis highlighted CPT1A (Carnitine Palmitoyltransferase 1A) as a prominent marker in the male cohort, consistently appearing in the top ranks. Other top-ranked genes included TXNIP (Thioredoxin Interacting Protein), which showed strong associations in both sexes, and PTGDR (Prostaglandin D2 Receptor). The global distribution of epigenetic associations across 28 cancer types was visualized using a bubble plot and a heatmap of HyPi-score intensities in beta value (Figure 5) and M value (Supplementary Figure S9). The bubble plot illustrates the broad impact of systemic lipid profiles, with the size and color intensity of each bubble representing the magnitude of the HyPi-score for specific lipid-CpG-cancer triplets. Triglycerides (TGY) emerged as the most prominent driver of epigenetic remodeling, showing consistently high scores across the majority of the 28 cancer types analyzed. Correspondingly, the heatmap in Figure 5 displays the ‘HyPi-score intensity’—a metric integrating both statistical significance and effect size—rather than raw methylation levels. This intensity map highlights distinct clusters of pan-cancer markers, where high-intensity regions (red) indicate robust links between dyslipidemia and clinical parameters (normal/tumor differences, survival, and clinical stage). Notably, master loci such as CPT1A and TXNIP exhibited peak intensities across diverse malignancies, suggesting that these genes act as central nodes in the lipid-driven metabolic reprogramming of the cancer epigenome.

### 3.4. Network Topology and Lipid Specificity

**Figure 6:**
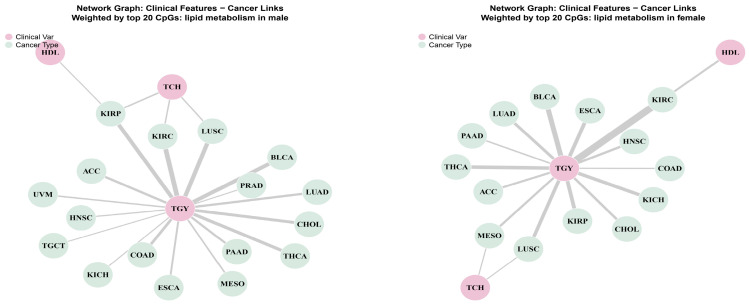
Network topology of lipid variables and cancer types. Network graphs illustrating the connectivity between lipid variables (orange nodes) and cancer types (sky-blue nodes). (**Left**) The male network shows TGY as a central super-hub connecting to gastrointestinal and renal cancers. (**Right**) The female network displays connectivity driven by TCH and HDL, linking to reproductive cancers. Edges represent shared epigenetic markers from the top 20 list.

The Network Graph of beta value (Figure 6) and M value (Supplementary Figure S10) elucidates the topology: TGY acts as a central “Super-Hub” connecting distinct cancer clusters, whereas HDL often shows opposing associations, reflecting its protective role. This distinction is quantified in the Variable Contribution analysis (Supplementary Figure S6), where TGY accounts for over 60% of the epigenetic prognostic score in digestive and renal cancers in males. In females, TCH plays a more significant role, validating the sex-specific impact of lipids on cancer biology.

## 4. Discussion

This study provides compelling multi-omics evidence that systemic lipid profiles are not silent bystanders but active participants in the cancer epigenome. The overwhelming dominance of TGY in our analysis supports the concept that hypertriglyceridemia provides an energy-rich environment that fuels cancer cell proliferation [32,33]. In particular, our pan-cancer analysis reconfirmed the critical role of CPT1A methylation as a master metabolic switch across diverse malignancies. CPT1A, identified as a master locus in our analysis (Table 1), is the rate-limiting enzyme in fatty acid oxidation (FAO) and plays a pivotal role in shuttling long-chain fatty acids into mitochondria for β-oxidation [34,35]. The epigenetic deregulation of CPT1A suggests that the body’s attempt to manage excess lipids might inadvertently activate metabolic pathways that cancer cells exploit for rapid proliferation and metastasis [36,37,38]. This finding underscores that CPT1A-mediated metabolic reprogramming is a shared hallmark of dyslipidemia-associated cancers, regardless of the tissue of origin.

Furthermore, the identification of TXNIP as a shared top marker (Table 1 and Supplementary Table S2) provides mechanistic insight into the interplay between glucose and lipid metabolism. TXNIP functions as a metabolic rheostat, linking glucose-lipid metabolic crosstalk with oxidative stress regulation [39]. In the context of tumorigenesis, TXNIP downregulation or epigenetic silencing—often driven by chronic metabolic stress—can impair redox homeostasis and promote glucose uptake, thereby facilitating a pro-tumorigenic environment [40]. Our results suggest that systemic dyslipidemia may drive the epigenetic remodeling of TXNIP, bridging the gap between systemic metabolic dysfunction and cellular oxidative stress in cancer cells.

Low HDL is a marker of systemic inflammation and impaired reverse cholesterol transport [27,41]. Our findings show that the epigenetic signature of low HDL overlaps with that of poor cancer prognosis. This implies that the loss of HDL’s anti-inflammatory and antioxidant functions contributes to a pro-tumorigenic microenvironment [11]. Unlike TGY, which appears to directly fuel tumor metabolism via FAO pathways, HDL’s protective role may be more closely linked to modulating the systemic immune landscape.

The strong link between Total Cholesterol (TCH) and female reproductive cancers highlights the intersection of lipid metabolism and endocrinology. Cholesterol serves as the essential precursor for steroid hormone biosynthesis, including estrogen and progesterone [42,43]. Moreover, cholesterol metabolites such as 27-hydroxycholesterol (27-HC) can function as selective estrogen receptor modulators (SERMs), directly binding to and activating estrogen receptors in breast cancer cells [44,45]. Thus, systemic dysregulation of cholesterol metabolism via epigenetic modifications may directly influence hormone-driven tumorigenesis in women, particularly in postmenopausal breast cancer, where aromatase inhibitor resistance has been linked to elevated cholesterol biosynthesis [46,47].

Our findings offer a new perspective on the debated anti-cancer effects of statins. While epidemiological evidence regarding statins has been inconsistent across different populations and cancer types [48], the lipid-related epigenetic remodeling identified in this study suggests a potential explanation. We propose that the clinical benefits of statins may extend beyond simple lipid-lowering; they may exert anti-cancer effects by normalizing specific DNA methylation patterns, such as those of CPT1A or TXNIP, that are distorted by chronic dyslipidemia [49]. This “epigenetic normalization” hypothesis could explain why certain patients derive significant survival benefits from statins while others do not, depending on their baseline epigenetic landscape.

## 5. Conclusions

By applying Hybrid Pi-score analysis integrating population-based (KoGES) and cancer genomic (TCGA) datasets, we demonstrated that dyslipidemia, particularly hypertriglyceridemia and low HDL cholesterol, exerts a profound and specific influence on the pan-cancer epigenome. The identification of master epigenetic loci, most notably CPT1A, provides mechanistic insight into how systemic lipid imbalances create a permissive environment for tumor progression via enhanced fatty acid oxidation [35]. These findings advocate for the aggressive management of lipid profiles as a crucial preventive and therapeutic strategy. Future studies should investigate whether lipid-lowering interventions can reverse these pro-oncogenic epigenetic signatures.

## Figures and Tables

**Table 1:** Characteristics of top lipid-associated CpG sites. This table lists the top 20 CpG sites selected based on their Hybrid Pi (HyPi) scores.

Probe ID	Count	HyPiPi *	Chr	Location **	Gene	Group	CpG Island	Sex
cg06943420	12	0.94	1	1566699	MMP23A	TSS1500	Island	M
cg06962177	15	−9.03	1	63785946			Island	M
cg04360793	13	−0.87	1	79472361	ELTD1	5′UTR	Island	M
cg18316974	16	−0.27	1	92947035	GFI1	Body	Island	M
cg06809252	14	2.95	1	110612044	ALX3	Body	Island	M
cg19693031	22	−6.86	1	145441552	TXNIP	3′UTR		M
cg09671258	13	1.31	1	180202530	LHX4	Body	Island	M
cg03900143	14	3.87	3	147111660	ZIC4	Body	Island	M
cg09142313	13	1.31	5	72677859			Island	M
cg01518607	14	−8.13	6	27235891				M
cg07788369	18	0.53	7	96619068	DLX6AS	Body		M
cg19695507	12	−0.2	10	13526193	BEND7	Body		M
cg05667348	12	−0.66	10	118892581	VAX1	Body	Island	M
cg02646491	11	−1.3	11	2890710	KCNQ1DN	TSS1500	Island	M
cg00574958	14	−1.21	11	68607622	CPT1A	5′UTR	N_Shore	M
cg17058475	28	1.32	11	68607737	CPT1A	5′UTR	N_Shore	M
cg18693395	12	−0.1	14	52735421	PTGDR	Body	Island	M
cg05232694	12	1.18	20	48809539			S_Shore	M
cg00495860	11	−2.89	21	38065524			Island	M
cg11442732	14	−7.12	X	133118088	GPC3	Body	Island	M
cg19250799	14	4.94	1	47910456			Island	F
cg19693031	17	−2.18	1	145441552	TXNIP	3′UTR		F
cg18623980	14	6.16	2	45240563			Island	F
cg10881615	13	0.84	2	69100119	BMP10	TSS1500		F
cg21185289	11	12.89	2	74743437	TLX2	3′UTR	Island	F
cg08189989	11	13.06	2	105459164			Island	F
cg25645064	13	3.89	3	147096130				F
cg08070771	14	1.23	3	147125758	ZIC4	TSS1500	N_Shore	F
cg06424065	25	−1.09	4	6247640			Island	F
cg03287574	16	4.29	5	1886956			Island	F
cg07565505	12	4.08	5	1887300			Island	F
cg26333652	11	2.18	5	2750758	IRX2	Body	Island	F
cg02081006	12	2.11	5	122430434	PRDM6	Body	N_Shore	F
cg24452128	12	1.7	6	10390919			Island	F
cg20431250	11	4.65	6	108492653	NR2E1	Body	S_Shore	F
cg18502142	13	9.25	7	96622709	DLX6AS	Body		F
cg03102442	11	1.11	8	125738432	MTSS1	Body	N_Shore	F
cg00574958	23	−0.64	11	68607622	CPT1A	5′UTR	N_Shore	F
cg17058475	22	0.8	11	68607737	CPT1A	5′UTR	N_Shore	F
cg12111714	11	6.46	13	26043472	ATP8A2	Body	Island	F

* Rounded to two decimal places. ** Genome assembly version: hg19.

## Data Availability

The R source code used for the analysis is available on GitHub (https://github.com/vitastarz-jpg/gimlab, accessed on 31 March 2026). The TCGA dataset can be download from the GDC Data Portal (https://portal.gdc.cancer.gov/, accessed on 31 March 2026). The KoGES dataset can be obtained through a process established by the Ministry of Health and Welfare of the Republic of Korea, following approval from the institution’s Institutional Review Board (IRB) (https://www.nih.go.kr/ko/main/contents.do?menuNo=300566, accessed on 31 March 2026).

## Supplementary Materials

The following supporting information can be downloaded at: https://www.mdpi.com/article/10.3390/cancers18071138/s1, Figure S1: Comprehensive Bioinformatics Pipeline for Methylation Data Preprocessing and Quality Control.; Table S1: Sensitivity Analysis Validating Robustness Against Sample Overlap; Figure S2: Computational Framework for Deriving the Hybrid Pi-score (HyPi) and Integrated HyPiPi; Figure S3: Global Connectivity Map of Epigenetic Links Derived from M-values; Figure S4: Multidimensional Radar Plots of M-value Epigenetic Landscapes; Figure S5: Scatter Plot Revealing Sexual Dimorphism in Lipid-Driven Epigenetic Links; Figure S6: Relative Variable Contribution Analysis across Clinical Endpoints; Figure S7: Directional Consistency Quadrant Plots Based on M-values; Figure S8: Venn Diagram of Top 20 Master CpGs for M-values; Table S2: Characteristics of Top Lipid-Associated CpG Sites Derived from M-values; Figure S9: Epigenetic Cluster Analysis of Cancer Types and Lipid Variables Using M-values; Figure S10: Network Topology of Lipid Variables and Cancer Types Derived from M-values.

## Abbreviations

The following abbreviations are used in this manuscript:

ASAS	Ansan–Ansung (Cohort)
BRCA	Breast Invasive Carcinoma
CITY	City (Cohort)
CPT1A	Carnitine Palmitoyltransferase 1A
CpG	Cytosine-phosphate-Guanine
DMR	Differentially Methylated Region
DNA	Deoxyribonucleic Acid
FDR	False Discovery Rate
HDL	High-Density Lipoprotein Cholesterol
HyPi	Hybrid Pi-score
KDCA	Korea Disease Control and Prevention Agency
KIRC	Kidney Renal Clear Cell Carcinoma
KoGES	Korean Genome and Epidemiology Study
LIHC	Liver Hepatocellular Carcinoma
NT	Normal vs. Tumor
OV	Ovarian Serous Cystadenocarcinoma
PAAD	Pancreatic Adenocarcinoma
PTGDR	Prostaglandin D2 Receptor
SAM	S-adenosylmethionine
TCGA	The Cancer Genome Atlas
TCH	Total Cholesterol
TGY	Triglycerides
TXNIP	Thioredoxin Interacting Protein
