# Supplementary material for: Systemic Dyslipidemia Drives Pan-Cancer Prognosis via Epigenetic Remodeling: A Hybrid Pi-Score Analysis
Source: Cancers (Basel). 2026 Apr 1;18(7):1138. doi: 10.3390/cancers18071138 (PMC13072190; doi:10.3390/cancers18071138)
Supplement: Supplementary file 1 [file cancers-18-01138-s001.zip › cancers-4126911-supplementary.pdf]

Supplementary Materials

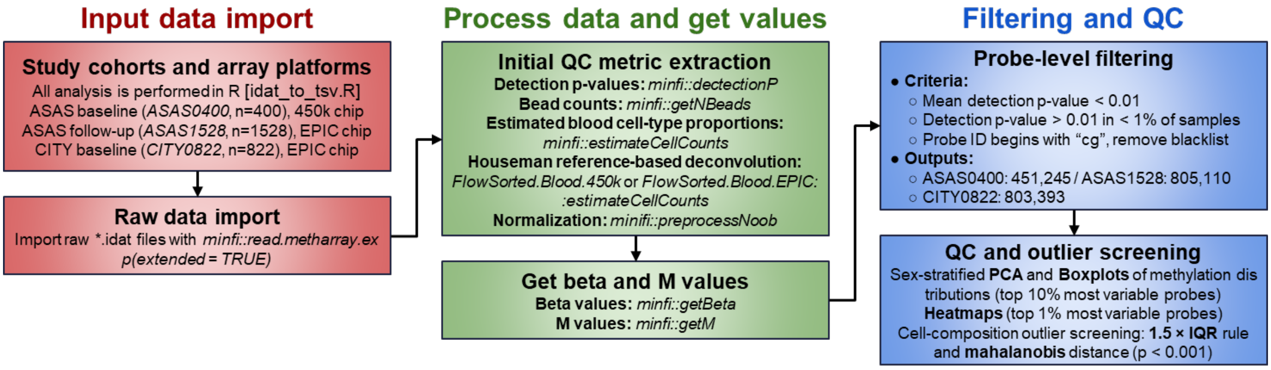

Figure **S1**. **Comprehensive Bioinformatics Pipeline for Methylation Data Preprocessing and Quality Control.** Flowchart diagram illustrating the stepwise computational workflow applied to the Illumina arrays (450K and EPIC) for the ASAS and CITY cohorts. The pipeline details raw IDAT import via the minfi package, probe-level filtering criteria, normalization procedures (Noob), cell-type proportion estimation using FlowSorted reference packages, and rigorous sample-level outlier screening based on the 1.5×IQR rule and Mahalanobis distance matrices prior to extracting exact Beta and M-values.

Table S1. Sensitivity Analysis Validating Robustness Against Sample Overlap. Table summarizing the strictly quantified statistical deviations resulting from the explicit exclusion of three overlapping individuals from the validation cohort (ASAS1528). The metrics evaluate both Beta and M-values across sexes, displaying the Mean Absolute Difference, the 99.9th percentile differences, and the near-perfect correlation of correlation vectors (r>0.99). The marginal differences confirm that the identified epigenetic signatures are stable and unconfounded by intra-individual longitudinal covariance.

	Mean absolute differences	99.9th percentile differences	Correlation of correlations
Per_Bvalue_M	0.000364361	0.003435632	0.9999465
Spe_Bvalue_M	0.000770812	0.002817251	0.999857146
Per_Bvalue_F	0.002298532	0.016639088	0.997958838
Spe_Bvalue_F	0.002015613	0.006405105	0.998986753
Per_Mvalue_M	0.000396458	0.003540199	0.999945542
Spe_Mvalue_M	0.000798816	0.00282819	0.999850665
Per_Mvalue_F	0.002386738	0.014183705	0.998101839
Spe_Mvalue_F	0.002077578	0.006483345	0.99894105

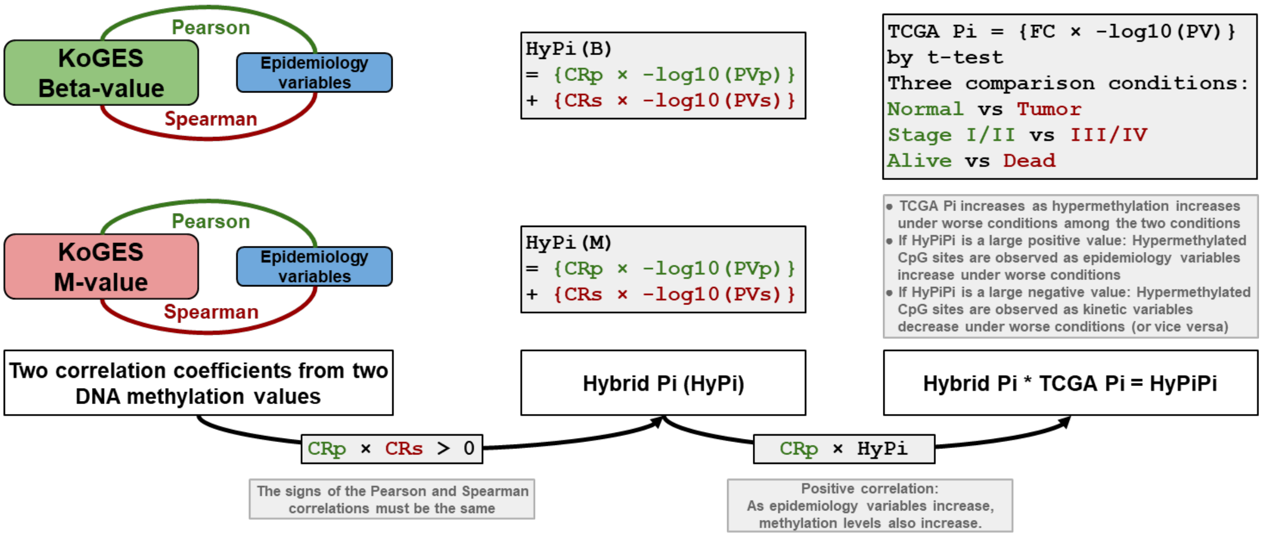

Figure S2. Computational Framework for Deriving the Hybrid Pi-score (HyPi) and Integrated HyPiPi. Schematic diagram demonstrating the parallel algebraic derivation of the Hybrid Pi-score. The model processes covariate-adjusted clinical lipid profiles and epigenome-wide variables (computed separately for Beta and M-values) via strict intersection of Pearson and Spearman correlations. It further illustrates the subsequent multiplication with TCGA-derived Pi-scores across three clinical contrast endpoints (Normal vs. Tumor, Alive vs. Dead, and Stage I/II vs. III/IV) to yield the final HyPiPi consensus marker.

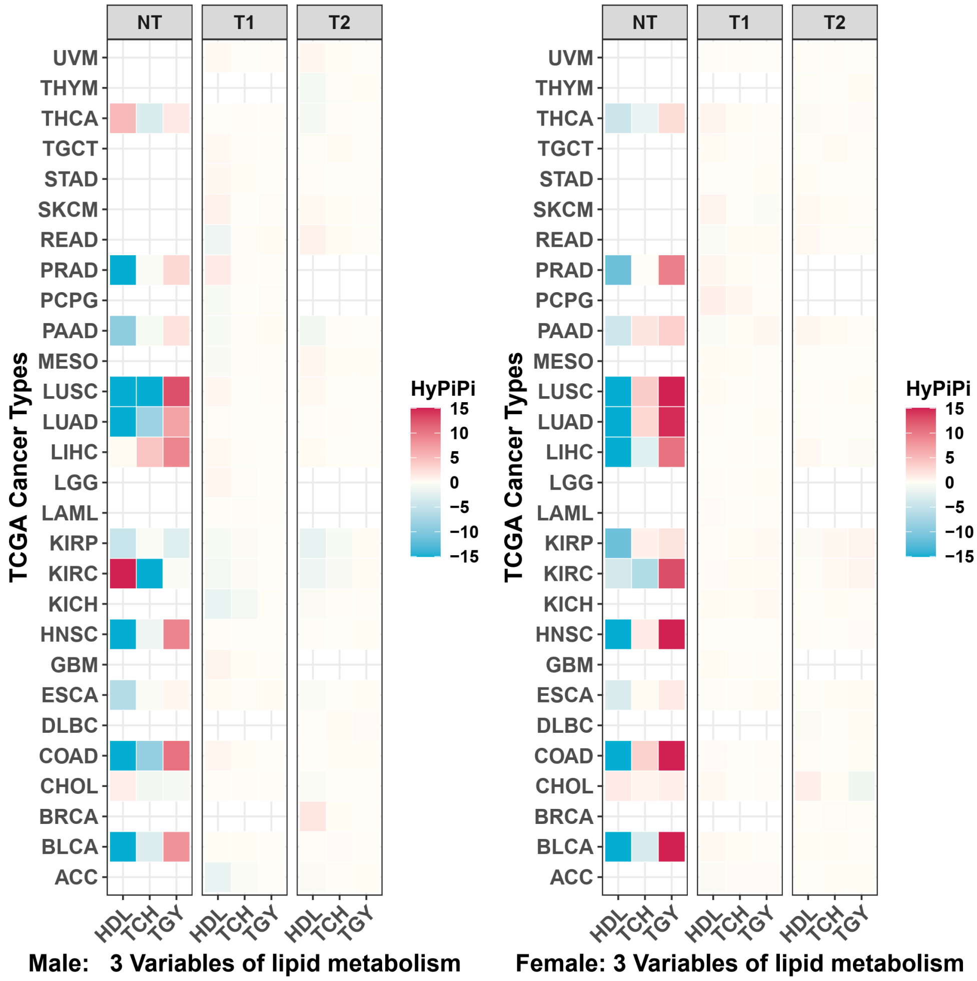

Figure S3. Global Connectivity Map of Epigenetic Links Derived from M-values. Faceted tile heatmaps illustrating the mean HyPiPi scores calculated

strictly from DNA methylation M-values across 32 TCGA cancer types. The left panel demonstrates the broad connectivity between lipid variables and metabolic cancers (e.g., LIHC, KIRC) in the male cohort, while the right panel reveals distinct, hormone-dependent cancer topologies in the female cohort. Color intensity scales with the magnitude of the epigenetic association.

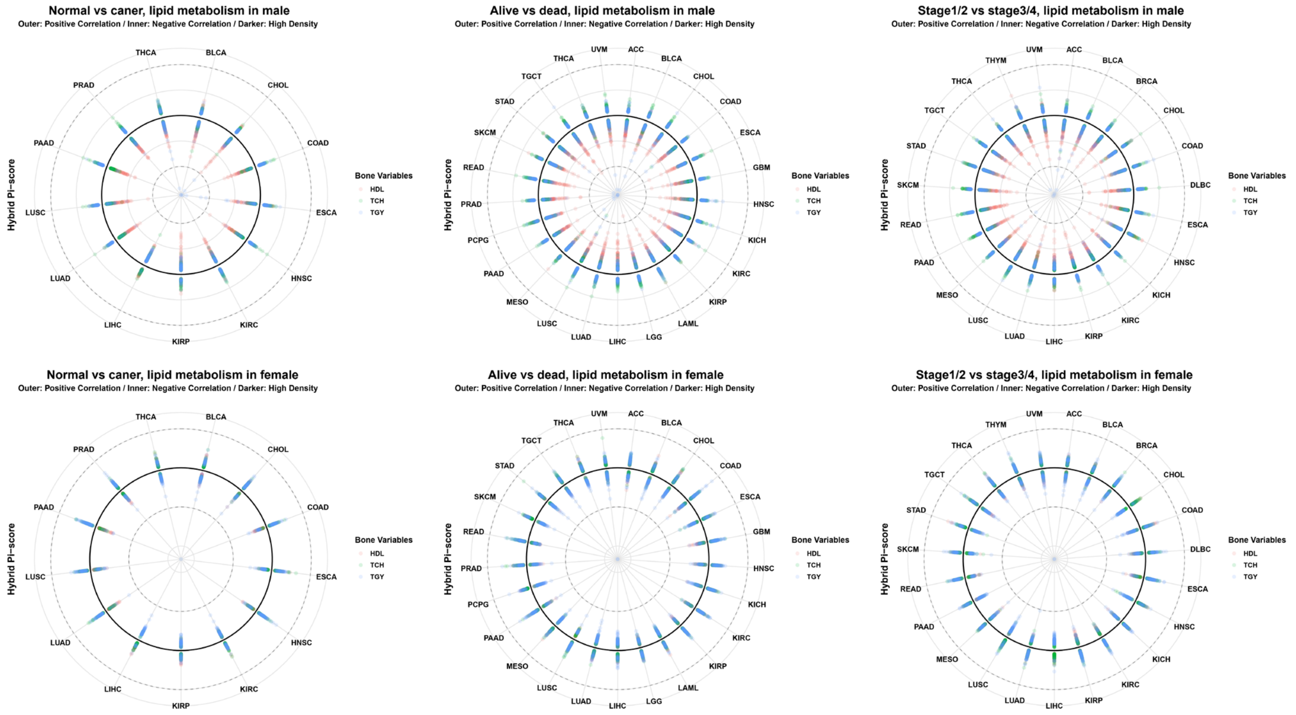

Figure S4. Multidimensional Radar Plots of M-value Epigenetic Landscapes. A comprehensive grid of radar plots mapping the Hybrid Pi-scores of lipid-associated CpGs (derived from M-values) across 32 pan-cancer cohorts. The radial expansions represent specific epigenetic clusters triggered by dyslipidemia stratified by sex (Top: Male; Bottom: Female) and evaluated across the three major clinical contrasts (Normal vs. Tumor, Alive vs. Dead, Stage I/II vs. Stage III/IV).

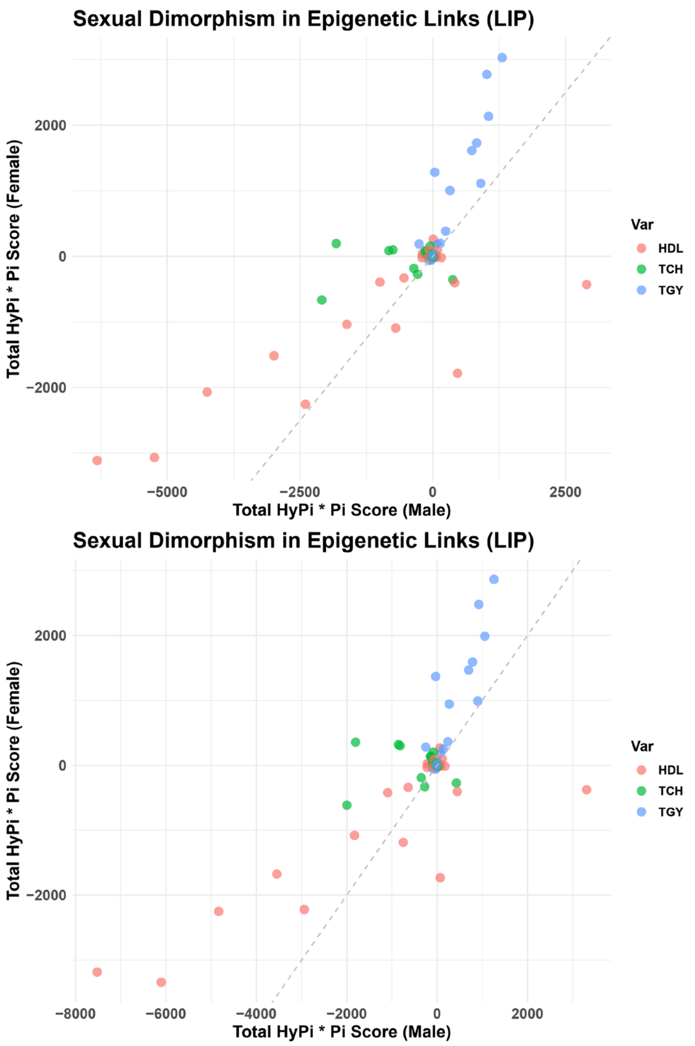

Figure S5. Scatter Plot Revealing Sexual Dimorphism in Lipid-Driven Epigenetic Links. Two-dimensional scatter plot comparing the Total HyPiPi scores between the male (x-axis) and female (y-axis) cohorts. Points are colored corresponding to specific lipid variables (HDL in red, TCH in green, TGY in blue). The orthogonal dispersion of the clusters underscores the distinct, sex-specific metabolic vulnerabilities embedded within the cancer epigenome.

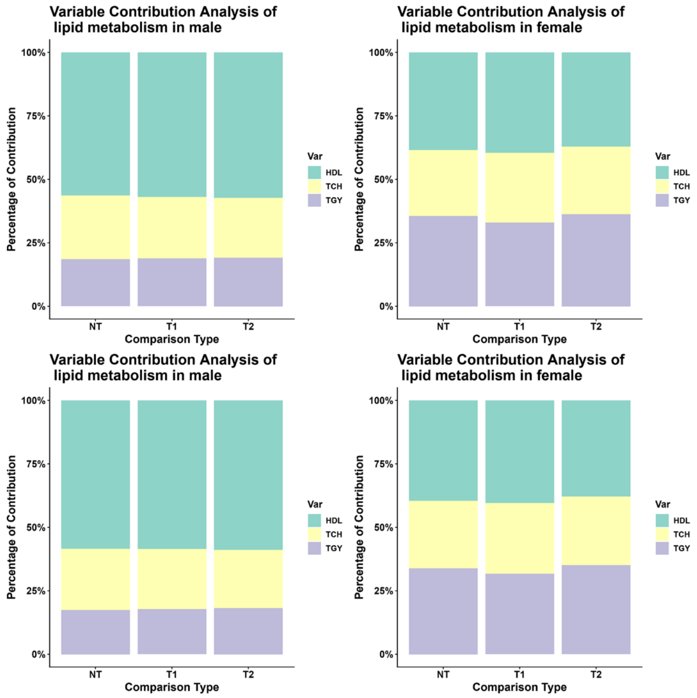

Figure S6. Relative Variable Contribution Analysis across Clinical Endpoints. Stacked bar charts delineating the proportional contribution (0–100%) of each specific lipid parameter (HDL, TCH, TGY) to the overall epigenetic prognostic score. Data are stratified by sex and segmented across the three primary comparative states (NT, T1, T2), highlighting the dominant predictive burden of Triglycerides (TGY) in determining tumor aggressiveness.

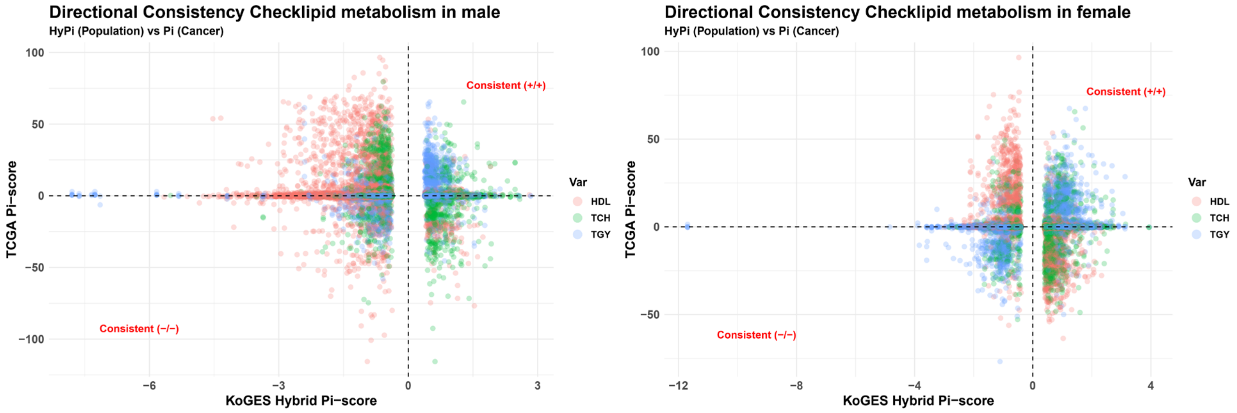

Figure S7. Directional Consistency Quadrant Plots Based on M-values. Scatter plots comparing the population-derived KoGES HyPi (x-axis) and the cancer-derived TCGA Pi-score (y-axis) computed strictly using M-values. Scatter distributions localized in the first (+/+) and third (-/-) quadrants provide empirical proof that the directionality of dyslipidemia-induced methylation in healthy individuals parallels the epigenetic reprogramming observed in high-mortality tumor groups.

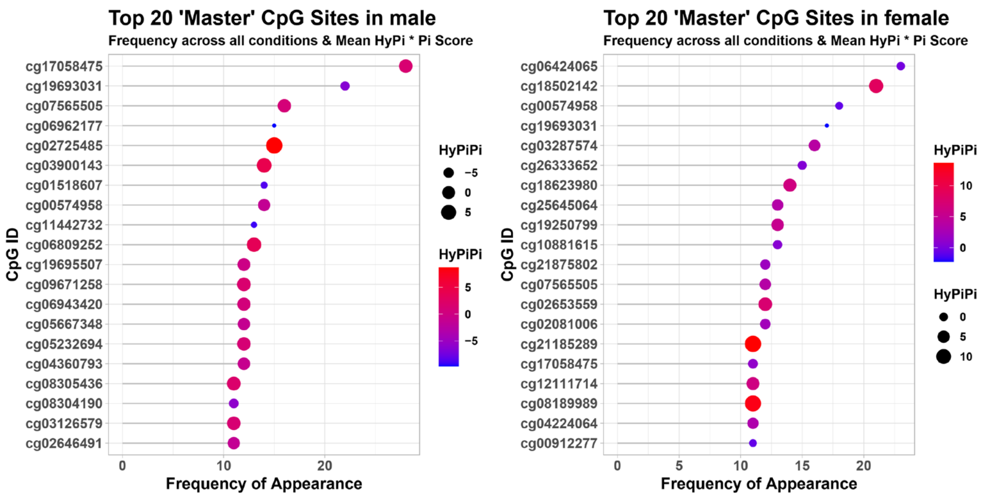

Figure S8. Venn Diagram of Top 20 Master CpGs for M-values. Venn intersection diagram quantifying the overlap of the top 20 master epigenetic regulatory loci—identified exclusively via M-value analysis—between the male and female pan-cancer cohorts. The visualization explicitly distinguishes sex-shared metabolic targets from sex-specific epigenetic drivers.

Probe ID	Count	HyPiPi *	Chr	Location **	Gene	Group	CpG island	Sex
cg06943420	12	0.62	1	1566699	MMP23A	TSS1500	Island	M
cg06962177	15	-9.73	1	63785946			Island	M
cg04360793	12	-0.95	1	79472361	ELTD1	5'UTR	Island	M
cg06809252	13	3.54	1	110612044	ALX3	Body	Island	M
cg19693031	22	-6.37	1	145441552	TXNIP	3'UTR		M
cg09671258	12	1.64	1	180202530	LHX4	Body	Island	M
cg03900143	14	4.21	3	147111660	ZIC4	Body	Island	M
cg07565505	16	0.83	5	1887300			Island	M
cg01518607	14	-8.57	6	27235891				M
cg19695507	12	-0.21	10	13526193	BEND7	Body		M
cg05667348	12	-1.15	10	118892581	VAX1	Body	Island	M
cg02646491	11	-1.33	11	2890710	KCNQ1DN	TSS1500	Island	M
cg00574958	14	-1.34	11	68607622	CPT1A	5'UTR	N_Shore	M
cg17058475	28	1.42	11	68607737	CPT1A	5'UTR	N_Shore	M
cg03126579	11	1.34	19	3822189	ZFR2	Body	Island	M
cg08305436	11	1.88	19	22806184			Island	M
cg08304190	11	-5.85	20	26188997	MIR663	TSS200	Island	M
cg05232694	12	1.15	20	48809539			S_Shore	M
cg02725485	15	8.69	X	30577300	CXorf21	3'UTR		M
cg11442732	13	-9.13	X	133118088	GPC3	Body	Island	M
cg19250799	13	5.34	1	47910456			Island	F
cg19693031	17	-2.26	1	145441552	TXNIP	3'UTR		F
cg21875802	12	1.93	2	45231382			Island	F
cg18623980	14	6.28	2	45240563			Island	F
cg10881615	13	0.69	2	69100119	BMP10	TSS1500		F
cg21185289	11	13.64	2	74743437	TLX2	3'UTR	Island	F
cg08189989	11	13	2	105459164			Island	F
cg25645064	13	3.6	3	147096130				F
cg02653559	12	7.44	3	147112081	ZIC4	Body	N_Shore	F
cg06424065	23	-0.15	4	6247640			Island	F

cg03287574	16	3.95	5	1886956			Island	F
cg07565505	12	3.7	5	1887300			Island	F
cg26333652	15	0.46	5	2750758	IRX2	Body	Island	F
cg02081006	12	2.3	5	122430434	PRDM6	Body	N_Shore	F
cg00912277	11	-0.79	7	55313376				F
cg18502142	21	8.67	7	96622709	DLX6AS	Body		F
cg00574958	18	-0.82	11	68607622	CPT1A	5'UTR	N_Shore	F
cg17058475	11	1.23	11	68607737	CPT1A	5'UTR	N_Shore	F
cg12111714	11	6.18	13	26043472	ATP8A2	Body	Island	F
cg04224064	11	3.22	14	36992233			Island	F

Table S2. Characteristics of Top Lipid-Associated CpG Sites Derived from M-values. Comprehensive catalog of the top 20 consensus CpG sites established via M-value based Hybrid Pi-score analysis. The table annotates the Probe ID, total frequency of significance across 84 analytical constraints (Count), integrated mean HyPiPi score, chromosomal coordinates mapping to genome build hg19, localized gene annotations, and respective functional genomic groupings delineated by sex.

* Rounded to two decimal places. ** Genome assembly version: hg19.

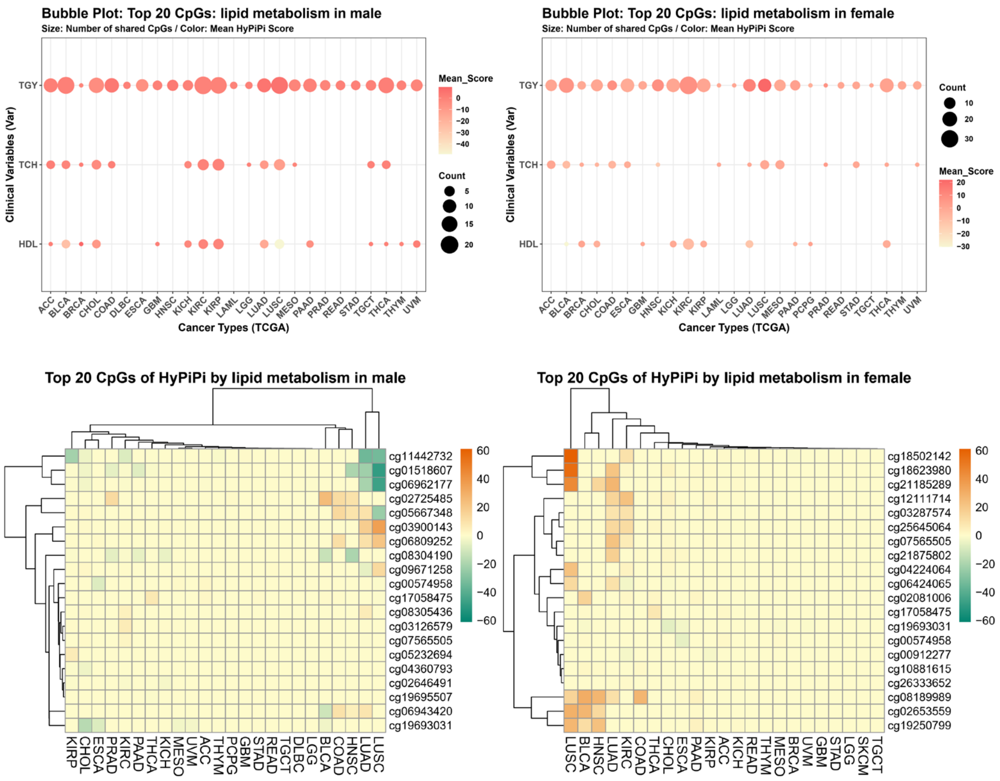

Figure S9. Epigenetic Cluster Analysis of Cancer Types and Lipid Variables Using M-values. Integrated visualization featuring bubble plots and hierarchical clustering heatmaps representing M-value data. The bubble plots illustrate the degree of connectivity (Count and Mean HyPiPi Score) between cancer types and lipid variables mediated by the top 20 master CpGs. The heatmaps correspondingly depict the condition-specific hyper- and hypomethylation profiles distinguishing protective (HDL) from pathogenic (TGY) lipid profiles across diverse malignancies.

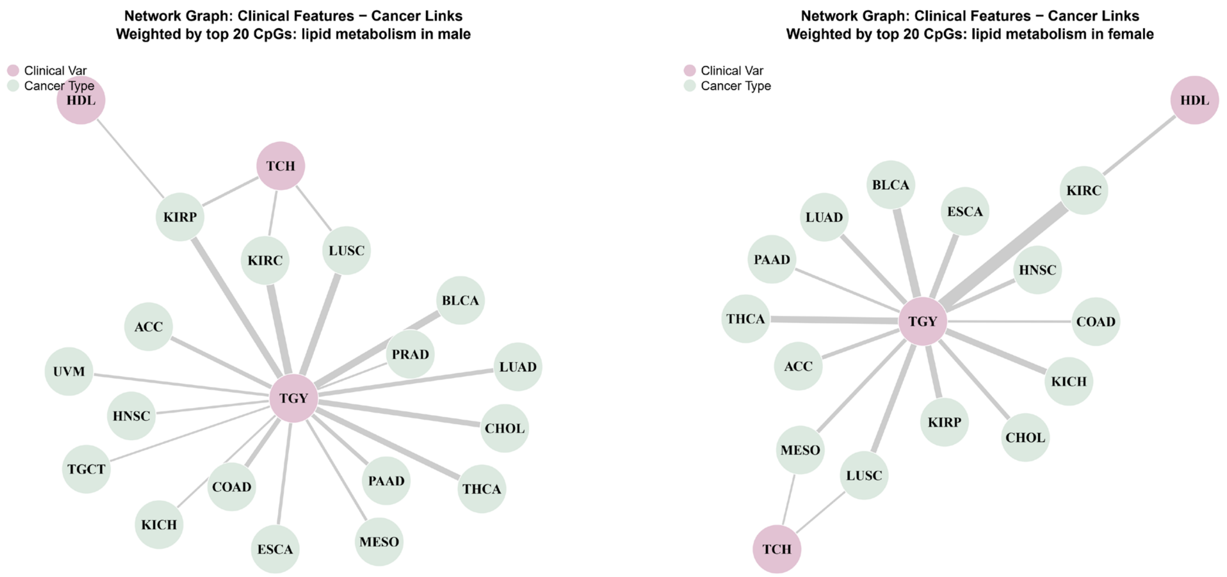

Figure S10. Network Topology of Lipid Variables and Cancer Types Derived from M-values. Network architecture graphs mapping the systemic epigenetic links utilizing M-values. The male topology network (left) identifies Triglycerides (TGY) as the dominant central super-hub orchestrating associations across gastrointestinal and renal cancers. Conversely, the female network (right) demonstrates an alternative structural connectivity heavily mediated by Total Cholesterol (TCH) and High-Density Lipoprotein (HDL) networks.
